# Salutogenesis: A sense of coherence and health among British military veterans exposed to impactful life challenges - the emergence of post-traumatic growth and positive deviance within a  life-story approach

**DOI:** 10.12688/f1000research.145255.1

**Published:** 2024-02-05

**Authors:** Richard Mottershead, Muhammad Arsyad Subu, Nabeel Al-Yateem, Wegdan Bani-Issa, Fatma Refaat Ahmed, Mini Sara Abraham, Jacqueline Maria Dias, Mustafa Muhamad Habeb, Nafi Alonaizi

**Affiliations:** 1 College of Health Sciences, Department of Nursing., University of Sharjah, Sharjah, Sharjah, United Arab Emirates; 2 Military Medical Services, Riyadh, Saudi Arabia

**Keywords:** Salutogenesis, Veteran, Health, Sense of Coherence, Post-Traumatic Growth, Positive Deviance.

## Abstract

**Background:**

This study sought to capture the perspectives and experiences of two sub-groups of military veterans, namely those who had previously been identified as offenders and those employed as practitioners within the criminal justice system in the United Kingdom.

**Methods:**

The lead author undertook narrative inquiry in the form of life story research. The life stories of 17 in-depth interviews were conducted across England and Wales, allowing for insight into the lived experiences of two life story trajectories of the health of military veterans. Existing literature on salutogenesis, health, post-traumatic growth, and positive deviance has also been investigated.

**Results:**

Life story interviews were transcribed verbatim and analyzed concurrently using thematic analysis to identify emergent themes. The researchers used thematic analysis as an analytical framework to allow descriptive themes from the literature on salutogenesis, health, post-traumatic, and positive deviance to be compared with those of life-story interviews.

**Conclusion:**

The study adopts a salutogenic approach, which suggests that an important indicator for a sense of coherence is enabled through the positive utilization of resilience through the reconstruction of military identity and experience. Notably, the concept of salutogenesis demonstrates the ability to draw from internal and external resources as circumstances require, to survive, and because of this, the participants’ health is maintained or improved. Hence, adaptation was notable through the coherence identified by the identity of being a veteran noted between the two subgroups and represents a continuous and dynamic process. The study suggests that the development of a sense of coherence is not restricted to the early decades of life but is a continuous process as contexts, personal circumstances, and opportunities arise. This later point is realized through the presence of post-traumatic growth and positive deviance, as they aid in the nurturing and development of a renewed sense of coherence via the known identity of military service and life.

## Introduction

The authors begin this paper by highlighting that most individuals (86%) leaving the Armed Forces transition into civilian life without difficulty and obtain employment which consequently allows for a positive contribution to society (Lord Ashcroft, 2014; Mottershead, 2019; Mottershead & Alonaizi, 2021). This paper explores the lived experiences of military veterans and themes that have become prominent features within the landscape of their life stories. The authors explored the concept of salutogenesis on the health care needs of these subgroups and their sense of coherence (SOC). The life stories voiced the struggles that the veterans encountered to maintain an identity within the presence of the stigma and associated shame of being identified as offenders. This study is reminiscent of the seminal work of Goffman (1963), who defined stigma as an attribute that is deeply discrediting to the individual. The life stories initially collected by the lead author provide insight into the veteran’s personal accounts of the impact of stigma and shame and the harm that is caused by the transitional friction encountered by the veteran and their family in being labelled as an offender that holds negative connotations to self-image and contrasts to having previously been identified as soldiers, sailors, or aircrews. In the study by Mottershead (2019), the life stories of the participants indicated that there was evidence that for some veterans, their life story trajectory found a sub-group to become segregated and isolated from a familiar veteran identity with few resources to survive the transition to a civilian life and new civilian identity. This exploratory qualitative study provides emancipatory evidence of the health and self of coherence and the impact of entering the criminal justice system (CJS) as offenders. According to McMurran, Khalifa, and Gibbon (2009), in England and Wales, the criminal justice process is delivered by a number of agencies that work collectively under the umbrella of the CJS. These include the Police, the Crown Prosecution Service, Her Majesty’s Court Service, the Youth Justice Board, Probation Service and the Prison Service. McMurran et al. (2009, p.2) further states that the ‘overall aims of the CJS is to detect and prevent crime, to rehabilitate and punish offenders and to support victims and witnesses of crime.

The aim of this study was to highlight evidence of post-traumatic growth and positive deviance. These two concepts provide inspiration and insight into how comradeship between military veterans is a cherished bond that appears to have been a source of motivation to address the health and social care needs of others. The participants also provided informed insights into the issues relating to veterans and sought to develop new systems needed to expand the knowledge base on the identification, diversion, and management of veterans and how to support those with mental health needs. The authors adopted a life story approach due to the evidence base that it constituted a practical way of gathering information about the veterans’ lives, which enabled others to step inside the personal world of the storyteller and experience other aspects of human experience ( Josselson and Lieblich, 1999; Ochberg, 1994). Braun and Clarke (2006, 2019) recommended that thematic analysis be adopted as part of multiple qualitative methods, as utilized within the methodology of this study. These researchers described thematic analysis as a process of visualizing and encoding qualitative research material through the formation of codes and themes, which will be discussed in the next subsection.

### The life story approach and thematic analysis

Health and social care research has long aligned itself with narrative approaches, with some notable seminal examples of life-story methods that will be highlighted in this section. In terms of studying veterans’ pathway into the CJS and specific health needs, life stories go beyond diagnosis, where a question-and-answer style is used to gather information ( O’Connor and Coleman, 1995). Gadd and Farrall (2004) emphasize that life stories also have a focus that is not associated with statistical risk-based studies on behavior. In relation to this study, this method of data collection enabled the veterans (participants) to position themselves in relation to discussing their health, how they deal with stress, stay well and improve their health, and maintain or develop resilience. Goodey (2000) advocates the use of narrative methods as a way of exploring how and why the individual has become dependent and, in relation to this study, how the individual has overcome and adapted to social structural forces that provided constraints or opportunities in the veterans’ life. Goodey (2000) explains that this approach is beneficial in that it supports the participant in identifying the relevant life choices that are significant in their story and therefore allows the authors to provide insight into the presence of post-traumatic growth and positive deviance within the narratives.

This approach is invaluable for comprehending veterans’ perspectives on their health and life trajectories. If the veteran’s life became absorbed with maladaptive behaviors unable to cope with the stresses of life, their social network diminished, and it was often left to statutory or charitable services to assist. This study included some veterans who had been incarcerated within the CJS due to offending behavior. According to McKendy (2006), the CJS exerts considerable powers of enforcement, but this study demonstrated that confinement went beyond physical realization as well as through discursive confinement as the participants experienced a diminished sense of coherence. The lead author sought to access these life stories to prevent restrictions on understanding and to ascertain meaning. When looking at the work of Maruna (2001), there is clear evidence that using life story methods with those identified as disenfranchised provides an opportunity for participants to reclaim their voice and develop a deeper understanding and awareness of themselves and their relationships with others. McKendy (2006) supports the approach of the authors, who ensure that this method is adopted so that the veterans can structure and interact with the interviewer and speak at length about their experiences. Leichtentritt and Arad (2005) explained that by using life stories, there is the potential to explore the social, emotional, interpersonal, health, accommodation, educational, and employment factors that have affected the life choices of veterans. In exploring the human element in the veterans account, the life story may prove invaluable in limiting the “us versus them” dichotomy between veterans having a dependency as opposed to them being perceived as positive contributors and protectors of national interests ( Williams, 2006; Mottershead, 2019). This is supported by Bochner (2015) and directed this study to include life story research to allow this methodology to empower participants to make sense of their lives. This approach was crucial to this study as placing the veterans’ experience of health and post-traumatic growth in a tangible form, which could then be transcribed and analyzed for meaning by the research team. Atkinson (2007) attests to the credibility of this approach, as it can create in-depth subjective meanings and enables the storyteller to impart life events in a way that they choose and want others to comprehend. This was important, as it facilitated the veterans to feel that they had ownership of their narratives and were able to articulate their life challenges so that meaning and understanding could be conveyed. Plummer (2001) stresses that although the researcher provides guidance, it is the participants’ voice that determines the frame of reference for the story, and the method captures the interactions between the individual and social world. According to Plummer (2001), this may include moments of indecision, turning points, confusion, and ambiguities common to everyday experience. At the commencement of this study, it was anticipated that there would be a wide variety and degree of complexity within the veterans’ life stories, specifically when exploring the impact of stigma, self-image, and evidence of post-traumatic growth. A comprehensive and adaptable methodological approach was required to support the meaning-making process of life stories; therefore, thematic analysis was selected.

As advised by Holloway and Todres (2003), this framework would need to be relevant to the research question of this study and was necessary to create an efficient method of analyzing the life stories of individual veterans. The life-story interviews were therefore analyzed using thematic analysis, as utilized by Boyatzis (1998) and Braun and Clarke (2006). This process of analyzing life stories was selected because of its flexible and straightforward technique that created an evidenced theoretical framework, which could provide insight into the lived experiences of veterans and their health and well-being. In this study, the lead author explored and revisited the data within the life story transcripts to produce pertinent health-related codes, then cross-referencing the whole data again to analyze coded extracts. Braun and Clarke (2006) stipulate that this constant moving back and forth is a necessity to establish the themes that establish meaning within the research. Table 1 illustrates the phases of analysis adapted from Braun and Clarke (2006, 2019).

**Table 1.** Phases of thematic analysis of the veteran life stories.

	Phase	Description of the process
1.	Familiarization with the Data	Reading and re-reading the life story transcripts, noting initial observations, thoughts and ideas.
2.	Generating Initial Codes	Coding pertinent features of importance within the data in a systematic approach across the entire data set (seventeen life story transcripts).
3.	Searching for Themes	Collating codes into potential health themes, gathering all relevant data to each potential theme.
4.	Reviewing Themes	Cross referencing themes back against all transcripts (seventeen) and the entire data set, establishing a ‘Thematic Map’ of the analysis.
5.	Refining the Thematic Map (see Figure 1.2 Adapted from Braun and Clarke, 2006)	Close inspection of previous stages to ensure that the Thematic Map provided an explanatory framework consistent with the transcribed life story. Further review, clarification and refinement of the Thematic Map.
6.	Writing the Analysis Adapted from Braun and Clarke (2006)	Selecting examples from the data to illustrate the themes and respond to the research question, analysing and interpreting the results by referring back to the study’s research aim and objectives, the literature and life stories.

This systematic approach adhered to the recommendations of Braun and Clarke (2006) and Savin-Baden and Major (2013), as this is a crucial procedure for identifying, analyzing, and producing patterns or themes found within the data. Thematic analysis was adopted because of its merits as an analytical device for constructing order and developing the main arguments of the topic for this study ( Guest et al., 2012). Braun and Clarke (2006) stated that one of the main benefits of thematic analysis is its flexibility, which is attractive when trying to identify themes with this sub-group of veterans.

**Figure 1.**
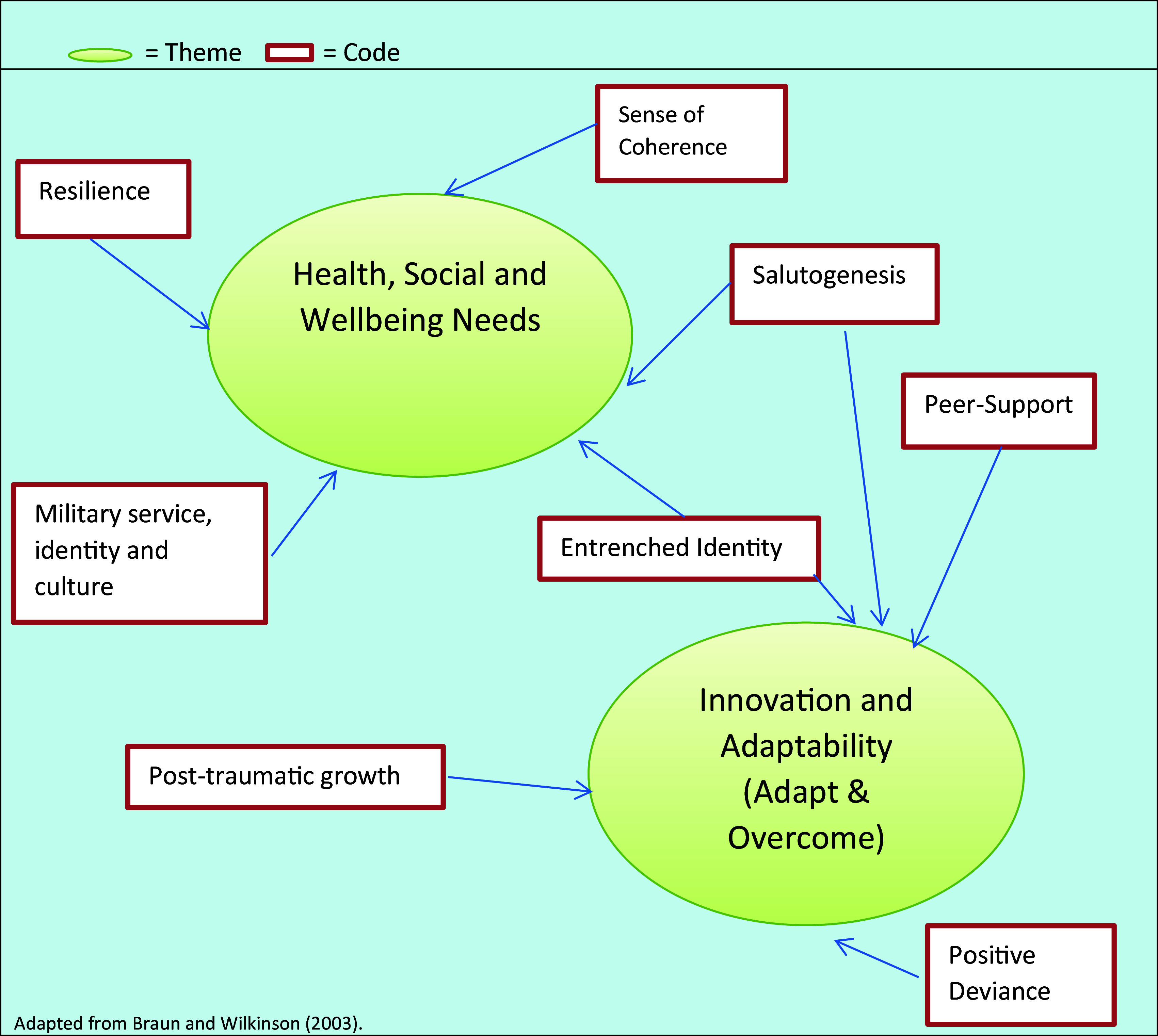
Thematic map of produced themes from the veteran life stories.

Etherington (2008) emphasized the need to be systematic when organizing life-story themes. Therefore, the lead author provided a chronological order of all the veterans’ stories within the life story trajectories of life before military service, during military service, and the period after discharge, thereby creating an aggregated story of all veterans’ stories. Etherington (2008) recommends that the focus on these stages of the life story trajectory allows the researcher to undertake numerous cycles of questioning, reflecting, rephrasing, theorizing, analyzing, and verifying the data, a quality assurance process that is evidenced to enhance the research findings. The researcher created a thematic map, as advocated by Braun and Clarke (2006), which created an image of the produced codes that galvanized the creation of the themes focused on within this study, as illustrated in Figure 1.

## Methods

### Ethical approval

This research study was submitted to the University of Chester’s Faculty of Health and Social Care Research Ethics Committee and was judged to be ethically robust and permission was granted to commence the data collection phase by a team of independent academics (RESC0513-413 – approved: 30.7.2013). All participants provided written informed consent to participate and for their anonymized data to be used for the purposes of this study.

### Data collection

Both subgroups were interviewed through the use of semi-structured interviews, but given the difference in their contact with the CJS, there was a need to create two separate sets of questions within an interview schedule ( Table 2).

**Table 2.** Interview Schedule – ex-offenders and practitioners.

Ex-offenders	Practitioners
• Please describe your life prior to entering the Armed Forces - Had you any involvement in the CJS prior to joining the Armed Forces? • What caused you to join the Armed Forces? • Please describe your career within the Armed Forces • What caused you to leave the Armed Forces? • What where your initial experiences when you re-entered ‘civvie street?’ • Do you believe you handled the transition well? What would your family and friends comments be on this question? • What support did you get from the Armed Forces during or leading up to this transitional phase? • What led you to becoming an offender within the criminal justice system? • Are there any parallels to your time within the Armed Forces and your time within the criminal justice system? • Do you maintain any links with your old Regiment/Corp? • Have there been any drastic changes to your life since leaving the Armed Forces? E.g. divorce • What support do you think could have made a difference in stopping you from becoming an offender since leaving the Armed Forces? • Do you know of any comrades who left the Armed Forces and became practitioners/professionals within the CJS? If so why do you think this happened? - How would you describe this individual? - How do they differ from you? Do you have any further points you would like to make?	• Please describe your life prior to entering the Armed Forces - Had you any involvement in the CJS prior to joining the Armed Forces? • What caused you to join the Armed Forces? • Please describe your career within the Armed Forces • What caused you to leave the Armed Forces? • What where your initial experiences when you re-entered ‘civvie street?’ • Do you believe you handled the transition well? What would your family and friends comments be on this question? • What support did you get from the Armed Forces during or leading up to this transitional phase? • What led you to a career in the criminal justice system? • Are there any parallels to your time within the Armed Forces and in working within the Criminal Justice System? • Do you maintain any links with your old Regiment/Corp? • Have there been any drastic changes to your life since leaving the Armed Forces? E.g. divorce • What has been the greatest support to you in allowing you to make the successful transition from the Armed Forces to becoming a practitioner within the criminal justice system? • Do you know of any comrades who left the Armed Forces and became offenders? If so why do you think this happened? - How would you describe this individual? - How do they differ from you? Do you have any further points you would like to make?

The process of collecting the stories from across the country took five months to complete. Interviews were conducted using a semi-structured format incorporating an interview schedule devised to incorporate themes identified from the literature review. Bachman and Schutt (2013) recommend this approach as it ensures that all dominant themes and main areas of interest are covered, guaranteeing consistency between interviews, whilst also maintaining sufficient flexibility to allow for unanticipated themes to be identified and discussed.

### Data analysis

A pilot interview before formal data collection took place where one veteran was interviewed using the life story method. Mason (2010) advises that a pilot study or interview should be included within a study’s research design, as this will create a crucial opportunity to analyse and review initial findings and to make forward decisions about whether there is a need to reassess the research question. As recommended by Bryman (2012), a digital recorder was used to record interviews, assisting greatly in the interview process and allowing both the participant and researcher to relax and converse more freely without the need for extensive notetaking, which can cause distraction and inhibit communication flow. One participant withdrew from the interview process due to on-going health issues. The interviews were undertaken in a variety of settings i.e., participant’s home, police station, university campus, probation offices, job centre, participant’s place of work, and hostel. At the time of the interviews only the lead author and the participants were present.

Within the study, seventeen individuals participated and gave their consent to allow their life stories to be shared. They all held the identity of a veteran, however eight of these veterans had since leaving the Armed Forces acquired roles within the CJS as practitioners. The remaining nine were defined within the CJS as offenders. This study defines the two sub-groups as follows:

1. Veteran practitioner, an individual who has performed military service for at least one day and drawn a day’s pay is identified as a veteran ( Royal College of Defence Studies, 2009). They had been an employee within one of the recognised institutions of the CJS.
2. Veteran offender, an individual who has performed military service for at least one day and drawn a day’s pay is identified as a veteran ( Royal College of Defence Studies, 2009). In relation to the label of ex-offender, the same individual had been charged with a crime, managed by the CJS and all sentences were spent.

As recommended by Silverman (2011), the recruitment of participants was based on snowball sampling. This study was supported by the Westminster Parliamentary Group on veterans in the CJS and the All Wales cross-party group on veterans and cadets. These groups were used as a starting point for identifying potential participants and other contacts emerged from initial contact with these organisations. Where necessary, access to participants was granted via internal organisational approval.

The various statutory and voluntary service providers who engaged with the researcher and study made initial contact with potential participants; this led to a number of practitioners within these services agreeing to be interviewed, or they knew of colleagues with a former military background who could meet the inclusion criteria.

The following sections within the results and discussion section, are formed from the emergent main and subthemes as identified within Figure 1 by the research team’s collective analysis of the data.

## Results and Discussion

### Salutogenesis: background

The researcher is a health professional and is aware that, in relation to the two separate life story trajectories, health cannot be understood in its narrowest sense. Health underpins ways of understanding how it may be achieved and maintained, even in the adverse circumstances of military transition. Such understandings include the concept of Salutogenesis, developed by Antonovsky (1979, 1987), to explain how some individuals utilize resources available to them in order to survive in adverse social conditions, and the associated construction ‘Sense of Coherence’ ( Antonovsky, 1979, 1987; Lindstrom & Eriksson, 2005), which informs ‘Asset based’ approaches to health and wellbeing ( Morgan & Ziglio, 2007). These constructions are important as they relate to uniformed personnel and how health is interpreted, understood, and impacts the ability to have resilience to life challenges.

Within the UK, it is worth acknowledging that around the time of the introduction of the National Health Service, health was highlighted by the United Nations as a subjective state of well-being within a psychosocial context, as well as an absence of illness ( UN Department of Public Info., 1948). The idea of a subjective health experience linked holistically to the physical, psychological, spiritual, and social well-being of individuals and populations has gained momentum since that time ( Mahler 1987; Lindstrom & Eriksson, 2006). These concepts are elaborated in the following section to explore the issues contributing to resilience. In addition, a review of the literature and exploration of dominant theoretical frameworks on the impact of health on veterans from life stories through the concept of salutogenesis was conducted.

### Salutogenesis in life story research

Salutogenesis, originally developed by Antonovsky, explains how some individuals utilize resources available to them to survive and thrive effectively in adverse social conditions ( Antonovsky, 1979, 1987). This concept also challenges the traditional understanding of health and illness, as represented by the medical model. Research that initially informed the concept of salutogenesis focused on how Israeli women maintained a good quality of health during incarceration in concentration camps of the Second World War ( Lindstrom & Eriksson, 2006). To reiterate, the ‘medical model,’ which emerged as a key concept for medical sociologists ( Conrad & Schneider 1992; van Teijlingen, 2005) has been challenged within contemporary medicine where a more holistic understanding has made inroads into the way in which we respond to some illness conditions. Engel (1977) argued that while the medical model constitutes a sound framework within which to understand and treat disease, it is not an appropriate framework within which to understand behavioral and psychological problems, as is clearly being exhibited with members of uniformed services. The acknowledgement that illness has psychological and social dimensions presents a challenge to Cartesian dualism, which underpins this model. For example, Merleau-Ponty (1964) argued that because human consciousness cannot be separated from the physical body, illness cannot be understood as a purely biological phenomenon. Instead, he stressed the importance of a holistic human being, in which the mind and body cannot be divorced. In privileging the centrality of the body in which the subject resides, he argued that the body has a dialogue with the world or, in other words, a reflective, absorbed engagement with the environment. When the body is ill, he argues that individuals must rethink their ability to engage with the world and provide movement, freedom, and creativity. Whereas the healthy body may be understood as transparent and taken for granted when it is ill, the harmony between the biological and lived body is disrupted and the difference between the two becomes apparent. Carel (2008) argued that health and illness can coexist in the same body, but that individuals do not always appreciate health until a body part ceases working, and then the focus of human consciousness becomes a lack of functionality on that part of the body. These may seem far removed from this article’s focus, yet reflexive review supports justification and linkage to causes of feelings of anger, frustration, and irrationality observed within the healthcare needs of uniformed services. For those making a transition between new identities, adaptation to an alternative construction of well-being must occur within the context of functionality. Moreover, this construction of health in its broadest sense is situated within a wider social context, which itself places constraints upon adaptation for veterans’ life post-conflict and those emergency service personnel whose working lives bring them to the front-line of conflict daily.

The study findings demonstrate how health, social and psychological aspects of the causality of ill-health for military veterans and emergency service personnel, mean it cannot easily be reduced to a few ‘bad eggs’ phenomena alone. This approach acknowledges how social and personal phenomena of a condition cannot be easily reduced to overarching dominant theory, and thus is apt to use as a framework within which to respond. In this study, an inclusive and holistic review of health in understanding emergent themes from the participants’ life stories offered insight into the structures of experience and consciousness of the participants’ physical, psychological, and social/practical support from military and emergency services. This resonates with Engel’s (1977) recommendation that an alternative model should consider not only the individual, but also the social contexts within which they live. He argued that a new biopsychosocial model which took into account all the factors which contribute to illness and patient-hood, (rather than giving primacy to biological factors alone) would make it possible to understand the individual’s ‘experience’ of disrupted well-being ( Carrió et al., 2004). This understanding, while not previously applied to the research undertaken on the health of uniformed services, would necessarily be situated within the context of an individual, their family, community, culture, and possibly their religious beliefs ( Gawinski et al., 2002). From the life stories, a positive transition from the former military identity could be viewed as achieving health, and the authors suggest that arguably it is not about having perfect physical health as much as coping and living well within current physical capabilities. Research findings would indicate that in relation to the subgroup of military veterans, they appear to have had health issues linked to maladaptive behavior, psychological trauma, and alcohol abuse, which has led to a deterioration in health and well-being, resulting in imprisonment ( Howard League of Penal Reform, 2011; Napo, 2009). The findings from the lead authors’ original study ( Mottershead, 2019) clearly highlight the importance of non-biological factors in participant well-being, leading some to ill-health and impoverished well-being. Indeed, interview discussions from that qualitative study highlighted issues around managing the transition and struggling to identify transferable skills, with some participants wishing they had never served in the Armed Forces. Likewise, frustrations were also evident for those within the emergency services who struggled with management who had no comprehension for the realities of the professional life and what constituted ‘normality.’ There was an emergent theme around the lack of understanding of what many participants felt the general public and the wider society had no comprehension of the lived experiences of those who had served their communities.

The authors believe that health and well-being cannot be conceptualized in the narrowest sense as a function solely of biology, which provides much scope for exploring how the quality of life may be achieved and maintained, even in adverse circumstances. It is in the context of this understanding that this study emphasizes that salutogenesis has an important role in creating insight into the health and uniformed services. A range of different theoretical concepts contribute to our understanding of salutogenesis ( Strumpfer 1990). These include Sense of Coherence ( Antonovsky, 1979, 1987; Lindstrom & Eriksson, 2005); Hardiness ( Kobasa, 1979; Kobasa, Maddi & Kahn, 1982); Learned Resourcefulness ( Rosenbaum & Palman, 1984); Potency ( Ben-Sira, 1985); and Stamina ( Colerick, 1985). These have been used to explore the concept of health as a resource for thriving and increasing quality of life ( Lindstrom, 1994). Specifically, Sense of Coherence SOC, which is integral to salutogenesis and thus the understanding of why some individuals are able to remain in relatively good health in conditions of adversity, while others are not ( Antonovsky, 1979, 1987). This concept relates to how individuals integrate into their society to utilize the resources available to them effectively to create and promote a good state of health, as can be seen within the veteran and emergency service sub-groups. Three concepts which underpin Sense of Coherence (SOC) are; ‘comprehensibility’ (where individuals can make sense of events), ‘manageability’ (where they feel they can take care of things) and ‘meaningfulness’ (where they really care about what happens) ( Antonovosky, 1979). These three concepts lie at the heart of Antonovosky’s salutogenic orientation, which represents a global orientation that can help understand how individuals respond to everyday external stressors and overcome stressful and challenging situations ( Antonovsky, 1996). Stressors include chronic stressors (such as disposable income), major life events (such as divorce), and acute daily hassles (time or support) ( Antonovsky, 1987).

Antonovsky (1979, 1987) argues that people create and build a sense of coherence over the first three decades of life. During this period, skills that operate flexibly and dynamically in any given environment were acquired. Given that the recruitment process into the military can commence at 16 years of age, it could suggest that immersion into a total institution (Goffman,1961) may impact the development of a sense of coherence, evidencing how military identity and culture impact development. This observation of development is significant as coherence creates skills to assist individuals in identifying the resources needed to address needs, related challenges, and source effective solutions to these, which promote positive well-being over the lifetime, as those within the emergency services can have extended working lives, whereas the military is predominantly constrained to 22 years.

In order to understand how Sense of Coherence might assist in promoting these individuals’ health-related behaviors, Johnson (2004) investigated its relevance in relation to health promotion with respect to self-esteem and locus of control. Johnson (2004) argued that a Sense of Coherence appears to have a strong and unique relationship with the pursuit of general health and a more tentative link to self-esteem or self-worth. A more stable locus of control was associated with an optimistic perspective towards coping with health challenges. Antonovsky (1993) had previously noted that Sense of Coherence, as a part of Salutogenesis, differed from ‘coping’ in the traditional sense. Because humans are continually surrounded by disease and associated stress, it is important to understand why certain people survive every day despite this situation ( Eriksson & Lindström, 2005). Hence, Sense of Coherence requires support from other theoretical frameworks such as ‘General Resistance Resources’ ( Griffiths et al., 2011) which purported that individuals may have instinctual drives to utilize financial, social, and cultural resources in order to create greater health stability for improved well-being ( Antonovsky, 1993). Antonovsky (1993) argued that greater General Resistance Resources (GRRs) means a more effective sense of coherence with respect to health promotion.

Having these resources increases the individual’s ability to deal with adversity and appears to be a feature lacking or present within the two sub-groups of uniformed services. Furthermore, Lindstrom and Eriksson (2006) identified a range of GRRs at the individual level, including physical (such as genetic resistance), material (such as money), cognitive (such as knowledge), attitudinal (such as self-esteem), and interpersonal (such as social capital) resources. At the social/structural level, they highlight welfare provisions and cultural traditions. While SOC is more of an orientation towards life, GRRs are biological, material, and psychosocial factors that enable individuals to perceive their lives as consistent, structured, and understandable ( Lindstrom & Eriksson, 2006).

Sense of Coherence has received considerable attention in the health care literature ( Langius-Eklof, Lidman & Wredling, 2009). For example, it has been explored in nursing interventions for cancer patients ( Delbar & Benor, 2001) and the treatment of young people at risk of developing mental disorders ( Ristkari et al., 2009). Delbar and Benor (2001) investigated whether the ability to cope depends on internal resources or can be increased through a structured nursing intervention–the Self-Care Approach. They assessed the impact of the Self-Care Approach on 48 cancer patients living at home, undergoing cancer treatment, including radiotherapy, chemotherapy, or both. These patients were compared with the matched control group. Measurement of coping ability with responsibility for symptom control through the SOC and Multidimensional Health Locus of Control Scales showed that the intervention group had a significant improvement in their SOC scores in three subcategories (comprehensibility, manageability, and meaningfulness). This means that the Self-Care Approach is associated with enhanced coping ability. Ristkari et al. (2009) examined self-reported psychopathology, adaptive functioning, and sense of coherence among adolescent boys in Finland. This quantitative study, using the Young Adult Self-Report (YASR) and Orientation of Life Questionnaire (SOC-13), showed a non-specific association between poor SOC and somatic complaints and a range of diagnosed psychiatric conditions.

A systematic review by Lindstrom & Eriksson (2006) reported that a sense of coherence emerges as a health-promoting resource that strengthens resilience and contributes to the development of a positive subjective state of health. Griffiths et al.’s (2011) qualitative study investigated the usefulness of a sense of coherence in understanding how (n=20) mental health service users dealt with problems they faced in their lives. The study identified various resistance resources for both concrete and relationship-oriented problem solving, suggesting that SOC may play an important role in coping with stressors in the rehabilitation/recovery process and thus contribute to mental health and psychosocial functioning within the general population ( Griffiths, 2009) and could have wider implications for uniform services.

Other social scientific theories have explored the process by which individuals, groups or societies use SOC to promote effective health management ( Klein, 2013). Research by Ben-Sira (1985) demonstrated that, for the participants of this study, a sense of individual potency increases the ability to buffer stressful issues and manage them effectively, as ordered, predictable, and meaningful within the context of health and well-being. It has been argued that high individual potency weakens the association between stress, coping, and illness, helping individuals thrive in the face of health-related adversity. Kobasa, Maddi and Kahn (1982) focused on individual ‘hardiness’ as an aspect of personality type, suggesting that individuals respond to stressful life events, exhibiting different degrees of commitment, control and challenge. They argued that individuals displaying more hardiness tend to involve themselves more readily in specific encounters and attempt to influence their outcome favorably rather than being helpless ( Seligman, 1972). Those with greater hardiness are also argued to be more able (and expected) to embrace change in circumstances as normal rather than static coping. It has been argued that in supporting the sense of coherence approach, hardiness increases the ability to expect and embrace change as the norm ( Kobasa, Maddi & Kahn, 1982).

It should be noted, however, that evidence for the hardiness theory was generated with respect to research on efficacy among business executives rather than on related behaviors associated with participants linked to this study, which means that its applicability to a sense of coherence is unproven ( Blaney & Ganellon, 1990). The emergence of adopting a bio-psychosocial model to explore and understand the life stories of military veterans’ exposure to life challenges has merit based on the success attributed to this approach, from simply not adhering to a dominant theoretical model. Mottershead and Ghisoni (2021), Mottershead (2022) and Mottershead and Alonaizi (2023) demonstrate variations in dominant treatment models for specific groups and the benefits of seeking theoretical models that are innovative and holistic. Similarly, focusing on veterans’ presence in the CJS via criminology and penological research alludes to the inclusion of other fields that can bring much-needed insight into this area of study.

It is essential to acknowledge that Salutogenic theory and SOC underpin health asset approaches ( Morgan & Ziglio, 2007). The notion of assets, or resources, as antecedents of health has been increasingly cited in the health promotion literature to explain social differences in health and wellbeing, but never to explore the meaning and understanding of military veterans’ involvement in the CJS. Charlton and White (1995) explain inequalities in health, including differences in health-related risk behaviors (as seen within the veteran community), in terms of the ‘margin of resources’ differentially available to individuals. Hence, an individual’s margin of resources, which is equal to their available resources less their essential needs, constitutes salutogenic factors associated with the optimum opportunity for improving health and well-being. Consequently, when analyzing the two veteran sub-groups of life stories, we can see that a broad definition of health and well-being allows for an understanding of the causality of one life story trajectory as a practitioner and the other as an offender.

The assets- or strengths-based approach is underpinned by understanding health as a resource for everyday life, rather than the object of living. From this perspective, health denotes social and personal resources as well as physical capabilities. In an asset- or strength-based approach, the strengths and resources of the individual and community are emphasized. Fundamental to this perspective is how the capabilities and characteristics of individuals and communities contribute to health and well-being, and as identified by Ashcroft (2014), these can be significant for a select portion of ex-service personnel entering civilian life. Health assets, defined as resources at the disposal of individuals and communities that protect against negative health outcomes and/or promote health status, can be social, financial, physical, environmental, or human resources ( Morgan & Ziglio, 2007). All prominent features within life stories that represent a submerged reef are ready to render those making the transition rudderless and ready for submersion into the CJS as an offender.

Although salutogenesis and its applications have received increasing attention from the research community, it should be noted that there is no evidence for the long-term sustainability of the Sense of Coherence and other applications of salutogenic theory with respect to public health ( Eriksson & Lindström, 2005). Outside the confines of this study, there is a lack of published research that further identifies the foundation of salutogenesis in the context of thriving and surviving for military veterans exposed to the CJS.

The 17 veteran participants involved in the study presented here drew on a range of resources to maximize personal well-being. Central to the identification and deployment of these resources was the objective of reconstructing a new civilian identity, allowing for a version of normality within their new lives post-discharge from the Armed Forces. In this reconstructed identity, they attended to the physical, psychological, and social aspects of their new civilian experience; for those unable to adapt, there was evidence of involvement in the CJS as an offender rather than as a practitioner.

The findings suggest that an important indicator for a Sense of Coherence is enabled through the utilization of resilience resources through which the reconstruction of a version of normality, mapping on to previous lives, is facilitated. Notably, the concept of salutogenesis concerns the ability to draw from internal and external resources as circumstances require, in order to survive, and because of this, it is not about having the static ability to move to a positive place and remain there ( Eriksson & Lindström, 2005). Hence, adaptation was notable through the coherence identified by the shared identity of being a veteran noted between the two subgroups and represents a continuous and dynamic process. As Eriksson and Lindström (2005) suggest, the development of a sense of coherence is not restricted to the early decades of life, but is a continuous process as contexts, personal circumstances, and opportunities arise. Given the age of recruitment into the Armed Forces, as highlighted in this study, there may be a need to further explore the impact of adolescents spent within a total institution on the development of a sense of coherence. Based on these points, the following section will highlight the studies and the researcher’s positionality and connect this epistemology simultaneously to acknowledge empowering and disempowering practices and policies towards and possible for veterans exposed to the CJS. The next section seeks to establish an understanding from the theoretical concept of salutogenesis and continues with the need to further investigate veteran identity and culture.

### Entrenched military identity: the dichotomy of identity and culture

Both subgroups of veterans described clear duality of identity. Among the practitioners, this was voiced within positive narratives of having achieved, and now in various guises, they reap the rewards of having been trained and served within the Armed Forces. There was a clear indication from this subgroup of participants (veterans) that having belonged to a larger community with a clear focus and purpose had created a lasting positive transferable skill set that had a positive influence, as evidenced within the life stories. As previously identified, most individuals (86%) leave the Armed Forces and enter civilian life without difficulty and obtain employment, which consequently makes a positive contribution to society (Lord Ashcroft, 2014; Mottershead, 2019). However, the same could not be said of the veteran offender subgroup. This group spoke about the shame and stigma of having been a serving member of the Armed Forces and is now being identified as an offender. There appeared to be a wish to have the first identity take priority, as this was discussed with pride and gave substance to their self-image, which was indefatigable and permanent, while the identity of the offender was spoken about in the hope of being transient and keen to have this label resigned to the past. Both subgroups spoke about belonging to a larger veteran community, a group within a society that they perceived as not fully comprehended by those outside of the group. Their life stories alluded to the belief that this was due, in part, to society not having shared attributes, a common value system, a unique culture, and a shared history. Cohen and Taylor (1990) and Clemmer (1958) explain that the replacement of a previous identity with one conducive to life within an institution can force the individual to become institutionalized and adopt traits associated with that environment.

Both sub-groups perceived that there was still a shared comradeship in belonging to the veteran community, and this bond was evident as there was a bond between the two sub-groups who would speak about their support and, at times, symbiotic relationships when the two would come into contact with each other within the criminal justice system. This feature has been explored and identified within research from the United States, first by Hollingshead (1946), who highlighted the struggles of returning G. I. in making the transmission back into their civilian lives, and later by Wortzel et al. (2009), who saw the support offered to veterans serving custodial sentences in the US by veterans employed as prison staff. This study highlighted the benefits of a shared lived experience in assessing and preventing suicide associated with shame and challenges linked to incarceration.

Within the life stories of the participants, there was a common thread of how military service led to the process of the creation of a reconstituted identity, formed through training, socialization, and contact with military culture. This reconstituted identity lends itself to allowing the individual to be assimilated into the entire institution of military life. This process is described by Goffman (1961), who described it as ‘disculturation’ involving the internalization of military culture with inculcation of habits, mannerisms and language as well as the breaking down of previously held moral boundaries and the assertion of an ‘adapt and overcome’ ethos. Goffman (1961) noted that this ethos was evident in the presence of confidence, courage, organization, and the suppression of fear. The life stories indicated that these traits were present within the veterans from both subgroups, but it was not clear whether these traits were created as a bi-product of military service or whether the participants already possessed these traits and were attracted to the Armed Forces as a career choice because of a lack of prospects. Research undertaken by McCulloch (2005) and Raffo et al. (2011) indicate that many recruits entering military service from socially and economically deprived areas struggle to meet basic eligibility requirements, have low academic achievement levels, poor health and fitness, histories of drug use, and criminal records. This may indicate that military service had a positive impact on traits, supporting the development of resilience, sense of coherence, and improved health during military service. This innovative ethos was a theme within the life stories, as they bore testimony to the development of schemes that had been born out of a need to improvise, adapt, and overcome a presenting challenge that it was felt had not been addressed by current policies and procedures. Indeed, this necessity to ‘overcome’ held within its discourse a concept of a need to work outside of previously held norms and to grasp upon a method to improve quality, safety, and access to healthcare inside and outside judicial provision.

### Improvise, adapt and overcome: post traumatic growth and positive deviance

The military veterans within this study were a unique population with previous exposure to various potentially traumatic and stressful life events, such as military training, deployment, combat, and the rigors of transition into the CJS as offenders as well as practitioners. A theme emerging from the life stories was the ability of these two subgroups to demonstrate post-traumatic growth. This was realized through meaningful psychological change after stressful and traumatic life events, as discussed by Zoellner and Maercker (2006), and was evident within the participants’ narratives as improved functioning and greater resilience. A relevant study by Feder et al. (2008) showed evidence of post-traumatic growth within a prison population, although this was during the war. A previous study by Boerner et al. (2017) provided some insight into the cautionary view that whilst post-traumatic growth may lead to positive psychological changes, it is also feasible to consider that it may be a dysfunctional attempt to manage and avoid the coping mechanisms that allow individuals to deal with stressful life events. There are two known studies from the UK that have focused on post-traumatic growth with veterans: Murphy et al. (2017) and Dyball et al. (2022), which have had a health focus with the latter study concluding that a moderate or large degree of post-traumatic growth among UK serving and veterans is associated with mostly positive health experiences, except for those with a diagnosis of post-traumatic stress disorder.

The life stories attested to evidence of post-traumatic growth for the veteran offender sub-group of psychological change resulting from the stigma and shame the individual initially experienced. The participants demonstrated the ability to relate to their veteran peers (offenders), a new understanding of their own personal strength, perceiving new possibilities in their lives that they focused on creating and establishing new innovative initiatives to support veterans now within the CJS as offenders or ex-offenders. The research team believes that this process not only demonstrates the presence of post-traumatic, but also demonstrates how this concept is focused on the action of positive deviance.

The participants demonstrated an effort to create and promote systems which surpassed conventional expectations outside of conventional norms; which indicated the presence of positive deviance. This concept is the observation that in most settings a few at risk individuals follow uncommon beneficial practices and consequently experience better outcomes than their neighbours who share similar risk ( Marsh et al., 2004).

Life stories contest the presence of deviant behavior and positive deviance. The latter appeared as attempts to establish new ways of working to support veteran ex-offenders and echo the military ethos of adapt and overcome. Positive deviance is defined as

“ *Positive deviance is the observation that in most settings a few at risk individuals follow uncommon beneficial practices and consequently experience better outcomes than their neighbours who share similar risk”.*

            
Marsh, Schroeder, Dearden, Sternin, and Sternin, (2004 p.1177)

A point articulated by the participants was that some behaviors encouraged within the military would be seen as actions that would classify them as deviant within civilian life. However, while this observation was made by the participants, there was a unilateral belief that the assessment and classification of deviant behavior was viewed as a necessity for this society, as it reinforced the social cohesiveness of its population by establishing rules to punish those that infringe and abuse these rules. Even ex-offenders appeared to appreciate the need for custodial boundaries. To this end, the veteran practitioners did not excuse the veteran ex-offenders’ actions but instead sought to establish systems that the research was based on the concept of positive deviance. This process within the life stories demonstrated the ability to stretch a norm by creating a morally correct action for the veterans and the community concerned. The shared veteran identity appeared to resonate between the two subgroups, and there was evidence of an ability to create effective veteran peer-support schemes that built on belonging to this veteran identity. This process appeared to aid in the elevation of frustrations for the participants while allowing for the progression of new improvised systems, adapted to overcome outdated practices towards veterans in the CJS, and demonstrating the presence of positive deviance. 

This concept, not fully explored in military research, explains that positive deviance has started to be identified within the field of international public healthcare and has been identified as a method to improve the quality and safety of healthcare ( Marra, 2010). This concept was present within both subgroups but was strongest within the veteran practitioners who appeared to adapt, overcome, and improve current processes with the explicit intention of supporting fellow veterans. Adamsky (2010) and Bassingthwaighte (2011) demonstrated that this scheme of working appeared to have a resonance enshrined within military training, which promoted the individual’s ability to improvise, adapt, and overcome. Dodge (1985) emphasized the need to progress past the overly negative conceptualization of deviance and instill a realization that individuals and/or their actions can be deemed as superior by virtue of surpassing conventional expectations. He argued that these positive deviants had surpassed their counterparts in sports, science, and politics because they operated outside of the perceived normal parameters ( Dodge, 1985). This ideological shift within sociological perspectives on the concept of deviance is significant in this study’s findings and demonstrates new knowledge when applied to military culture. With regard to the veterans within both subgroups, there appeared to be evidence of this approach as their actions sought to prevent and reduce harm by striving to negate possible harmful outcomes. Tuhus-Dubrow (2009) identifies positive deviants as exemplary in practice, either as individuals or part of a collective group. The presence of evolving strategies outside of normal practices was evident and demonstrated the presence of positive deviance, as the participants created multiple schemes to help aid veterans within the CJS, including those with specific health and social care needs.

One such initiative was the Veterans in Custody Support Scheme (VICS). One participant led the development of this scheme to identify offenders who have served at the earliest possible opportunity, with identification of having served in the Armed Forces occurring within police custody or within the prison service ( Phillips, 2014).

The participants’ attempts to establish and disperse the scheme were admirable, but they often faced hardships in their attempts to provide support. Fossey et al. (2017) explain that although the scheme is not mandatory for prisons and has no designated funding, it continues to spread across the prison service. Indeed, participants in the study were actively involved in supporting the scheme by establishing it within police custody suites, and prisons were employed. Once veterans have been identified, the scheme refers to them as community ex-service organizations for resettlement assistance ( Phillips, 2014).

In relation to well-being and mental health, this process of transition into the CJS meant a realization that, for some innovative schemes, focusing on peer support through a shared ex-military service identity aided in the start of rehabilitation. This unexpected benefit through forced capitulation with the CJS created the opportunity for some within this study to identify unmet social and health needs and commence rehabilitation and redemption for mistakes. The thematic analysis of the life stories provided evidence of mental fall-out through stigmatization and shame of being identified as an offender, which then had consequences on the participants’ sense of coherence. However, while the process of progressing through the CJS was traumatic, there was acknowledgement that this environment could at times be a reprieve from the chaos that had led them to become offenders through their maladaptive behaviors.

### Limitations of the study

The findings of this study do not claim generalizability, having emerged from one country and one homogeneous sample (albeit two sub-groups) providing their subjective lived experiences, together with the researcher’s own interpretation of them. Undoubtedly, the researcher’s own subjectivity, lived experiences, and research interests may have influenced the interpretation of these findings, as highlighted by Blaikie (2007). However, precautions were taken to limit the impact of this bias, where possible, by adhering to a clear and robust methodological framework and review of the original research by a team of researchers. Given the modest deficit of knowledge in this area, this study provides insight and contributes to new knowledge.

## Conclusion

This study can be utilized to establish a way of knowing within two realities, which has created knowledge about the experiences of individual veterans, to represent the position of stakeholders, and to go beyond the generalization of policies and practices relating to this group’s experiences. This developing issue has been overlooked, perhaps due to financial shortcomings, finite resources, and issues relating to stigma and shame forcing a camouflaged subgroup with specific health and social care needs. This study suggests that there is a need to establish a new perspective and understanding of the impact of being a veteran with challenges around the specific needs of this group, which merit further expansion of bespoke health and well-being services and treatments.

Just over ten years ago, practitioners began to express concern that a problem existed as they recognized fellow veterans entering the CJS as offenders. This realization allowed for synergy between post-traumatic growth that illuminated opportunities for paradigm shifts in individuals’ thinking and the establishment of new practices and policy alignment. Undeterred by budgetary confines and resources, the participants began to engage in positive deviance to use the innovation and knowledge of total institutions to engineer solutions in the establishment of systems to identify this vulnerable group. The authors have utilized the veteran’s own life stories and experiences to ensure that their voices were heard. It is hoped that this act will aid in understanding the stigma and shame associated with many with the eroded veteran identity due to a custodial sentence, as well as health needs that may affect the individual’s sense of coherence, as understood through the study’s salutogenic approach. The presence of post-traumatic growth is testimony to the resilience of this subgroup. Likewise, the establishment of innovative practices and procedures realized through the concept of positive deviance is further evidence of this subgroup’s ability to overcome adversity, demonstrate compassion through comradeship, and attempt to prevent cyclical problems related to a diminished sense of coherence and health through the capacity to cope with the stresses of life, to stay well, and to maintain good physical and mental health.

## Data Availability

Data cannot be shared publicly due to potentially identifiable information in the transcripts. Data will be made available to reviewers and researchers via the corresponding author following approval from participants when and if possible.

## References

[ref1] Adamsky D : *The Culture of Military Innovation: The Impact of Cultural Factors on the Revolution in Military Affairs in Russia, the US, and Israel.* Stanford University Press;2010.

[ref89] Antonovsky A : *Health, stress and coping.* San Francisco: Jossey-Bass;1979.

[ref2] Antonovsky A : *Unravelling the mystery of health. How people manage stress and stay well.* San Francisco: Jossey-Bass;1987.

[ref3] Antonovsky A : The structure and properties of the sense of coherence scale. *Social Science Medicine.* 1993;36:725–733. 10.1016/0277-9536(93)90033-Z 8480217

[ref4] Antonovsky A : The salutogenic model as a theory to guide health promotion. *Health Promot. Int.* 1996;11:11–18. 10.1093/heapro/11.1.11

[ref5] Ashcroft M : The veterans transition review. 2014 February [cited 2021 August 11]. Reference Source

[ref6] Atkinson R : The life story interview as a bridge in narrative inquiry methodologies. Clandinin D , editor. *Handbook of narrative inquiry: mapping a methodology.* Thousand Oaks, California: Sage Publications;2007; pp.224–245.

[ref7] Bachman RD Schutt RK : *The Practice of Research in Criminology and Criminal Justice.* 5th ed. London: Sage Publications;2013.

[ref8] Bassingthwaighte M : *Adaptive Campaigning Applied: Australian Army Operations in Iraq and Afghanistan.* 2011; vol.55; pp.39–45.

[ref9] Ben-Sira Z : Potency: A stress-buffering link in the coping-stress disease relationship. *Soc. Sci. Med.* 1985;21(4):397–406. 10.1016/0277-9536(85)90220-5 4049011

[ref10] Blaikie N : *Approaches to social enquiry: advancing knowledge.* Boston: Polity Press;2007.

[ref11] Blaney PH Ganellon RJ : Hardiness and social support. Sarason BR Sarason IG , editors. *Social Support: An interactional view.* New York: Wiley;1990. (pp.297–318).

[ref12] Bochner A : Coming to Narrative: A personal history of paradigm change in the human sciences. *South Commun. J.* 2015;80(4). 10.4324/9781315432090

[ref13] Boerner M Joseph S Murphy D : A theory of reports of constructive (real) and illusory post-traumatic growth. *J. Humanist. Psychol.* 2017;60:384–399. 10.1177/0022167817719597 Google Scholar

[ref14] Boyatzis RE : *Transforming qualitative information: Thematic analysis and code development.* Thousand Oaks, CA: Sage;1998.

[ref15] Braun V Clarke V : Using Thematic Analysis in Psychology. *Qual. Res. Psychol.* 2006;3(2):77–101. 10.1191/1478088706qp063oa

[ref16] Braun V Clarke V : Reflecting on reflexive thematic analysis. *Qual. Res. Sport Exerc. Health.* 2019;11(4):589–597. 10.1080/2159676X.2019.1628806

[ref17] Bryman A : *Social Research Methods.* 4th ed. Oxford: Oxford University Press;2012.

[ref19] Carel HH : *Illness. Art of Living.* Stocksfield: Acumen Publishing Limited;2008.

[ref20] Carrió F Suchman A Epstein R : The Bio-psychosocial Model 25 Years Later: Principles, Practice, and Scientific Inquiry. *Ann. Fam. Med.* 2004;2(6):576–582. 10.1370/afm.245 15576544 PMC1466742

[ref21] Charlton B White M : Living on the margin: a salutogenic model for socio-economic differentials in health. *Public Health.* 1995;109(4):235–243. 10.1016/S0033-3506(95)80200-2 7667487

[ref22] Clemmer D : *The Prison Community.* New York: Holt, Rinehart and Winston;1958.

[ref23] Cohen S Taylor L : Time and the Long-term Prisoner. Hassard J , editors. *The Sociology of Time.* London: Palgrave Macmillan;1990. 10.1007/978-1-349-20869-2_12

[ref24] Colerick EJ : Stamina in later life. *Soc. Sci. Med.* 1985;21(9):997–1006. 10.1016/0277-9536(85)90421-6 4081826

[ref25] Conrad P Schneider JW : *Deviance and Medicalization: From Badness to Sickness.* Temple University Press;1992;266–271.

[ref26] Delbar V Benor DE : Impact of a nursing intervention on cancer patients ability to cope. *J. Psychosoc. Oncol.* 2001;19:57–75. 10.1300/J077v19n02_04

[ref27] Dodge DL : The Over-Negativized Conceptualization of Deviance: A Programmatic Exploration. *Deviant Behav.* 1985;6:17–37. 10.1080/01639625.1985.9967657

[ref28] Dyball D Taylor-Beirne S Greenberg N : Post-traumatic growth among UK military personnel deployed to Iraq or Afghanistan: data from phase 3 of a military cohort study. *BJPsych Open.* 2022 Sep 23;8(5):e170. 10.1192/bjo.2022.570 36148897 PMC9534878

[ref29] Engel GL : The need for a new medical model: A challenge for biomedicine. *Science.* 1977;196(4286):129–136. 10.1126/science.847460 847460

[ref30] Eriksson M Lindström B : Validity of Antonovsky’s Sense of Coherence Scale: A Systematic Review. *J. Epidemiol. Community Health.* 2005;59:460–466. 10.1136/jech.2003.018085 15911640 PMC1757043

[ref31] Etherington K : Working with traumatic stories: from transcriber to witness. *Int. J. Soc. Res. Methodol.* 2008;10:85–97.

[ref32] Feder A Southwick SM Goetz RR : post-traumatic growth in former Vietnam prisoners of war. *Psychiatry.* 2008 Winter;71(4):359–370. 10.1521/psyc.2008.71.4.359 19152285

[ref33] Fossey M Cooper L Godier L : A pilot study to support veterans in the criminal justice system. *Final Report.* 2017. Reference Source

[ref34] Gadd D Farrall S : Criminal careers, desistance and subjectivity: interpreting men’s narratives of change. *Theor. Criminol.* 2004;8(2):123–156. 10.1177/1362480604042241

[ref35] Gawinski B Bennett P Rousseau S : A bio-psychosocial model of training in abortion care. *Fam. Syst. Health.* 2002;20(4):439–446. 10.1037/h0089512

[ref36] Goffman E : *Asylums: Essays on the social situation of mental patients and other inmates.* London: Penguin Books;1961.

[ref37] Goffman E : *Stigma.* Englewood Cliffs, NJ: Prentice Hall;1963.

[ref38] Goodey J : Biographical lessons for criminology. *Theor. Criminol.* 2000;4(4):473–498. 10.1177/1362480600004004004

[ref39] Griffiths CA Ryan P Foster JH : Thematic analysis of Antonovsky’s sense of coherence theory. *Scand. J. Psychol.* 2011;52(2):168–173. 10.1111/j.1467-9450.2010.00838.x 20626705

[ref40] Griffiths CA : Sense of coherence and mental health rehabilitation. *Clin. Rehabil.* 2009 Jan;23(1):72–78. 10.1177/0269215508095360 19114439

[ref41] Guest G MacQueen KM Namey EE : *Applied Thematic Analysis.* London: Sage Publications;2012.

[ref42] Hollingshead AB : Adjustment to military life. *Am. J. Sociol.* 1946 Mar;51:439–447. 10.1086/219855 21014486

[ref43] Holloway I Todres L : The status of method: flexibility, consistency and coherence. *Qual. Res.* 2003;3(3):345–357. 10.1177/1468794103033004

[ref44] Howard League for Penal Reform: *Report of the Inquiry into former Armed Forces Service Personnel in Prison.* London: Howard league for penal reform;2011.

[ref45] Johnson M : Approaching the salutogenesis sense of coherence: The role of the ‘active’ self-esteem and coping. *Br. J. Health Psychol.* 2004;9:419–432. 10.1348/1359107041557057 15296687

[ref46] Josselson R Lieblich A : *The Narrative Study of Lives.* Newbury Park, CA: Sage;1999.

[ref47] Klein C : Social Capital or Social Cohesion: What Matters For Subjective Well-Being? *Soc. Indic. Res.* 2013;110(3):891–911. 10.1007/s11205-011-9963-x Reference Source

[ref48] Kobasa SC : Stressful life events, personality and health: an inquiry into hardiness. *J. Pers. Soc. Psychol.* 1979;37(1):1–11. 10.1037/0022-3514.37.1.1 458548

[ref49] Kobasa SC Maddi SR Kahn S : Hardiness and health: A prospective study. *J. Pers. Soc. Psychol.* 1982;42(1):168–177. 10.1037/0022-3514.42.1.168 7057354

[ref50] Langius-Eklof A Lidman K Wredling R : Health related quality of life in relation to sense of coherence in a Swedish group of HIV infected patients over a two year follow-up. *AIDS Patient Care STDs.* 2009;23(1):59–64. 10.1089/apc.2008.0076 19063712

[ref51] Leichtentritt R Arad B : Young male street workers: life histories and current experiences. *Br. J. Soc. Work.* 2005;35(4):483–509. 10.1093/bjsw/bch192

[ref52] Lindstrom B : The essence of existence. Nordic School of Public Health, NHV-Report 1994:3, Goteborg. 1994.

[ref53] Lindstrom B Eriksson M : Professor Aaron Antonovsky (1923-1994) the father of salutogenesis. *J. Epidemiol. Community Health.* 2005;59:440–442. 10.1136/jech.2005.034777 15911636 PMC1757059

[ref54] Lindstrom B Eriksson M : Contextualising salutogenesis and Antonovsky in public health development. *Health Promot. Int.* 2006;21(3):238–244. 10.1093/heapro/dal016 16717056

[ref81] Mahler H Kohler L , editors. *Public Health: A Nordic Perspective.* Goteborg (in Swedish): Nordic School of Public Health;1987; Volume2.

[ref55] Marra AR : Positive deviance: a new strategy for improving hand hygiene compliance. *Infect. Control Hosp. Epidemiol.* 2010;31(1):12–20. 10.1086/649224 19925270

[ref56] Maruna S : *Making good: how ex-convicts reform and rebuild their lives.* Washington, D.C.: American Psychological Association;2001. 10.1037/10430-000

[ref57] Marsh DR Schroeder DG Dearden KA : The power of positive deviance. *Br. Med. J.* 2004;329(7475):1177–1179. 10.1136/bmj.329.7475.1177 15539680 PMC527707

[ref82] Mason J : *Qualitative Researching.* 2nd ed. London, Mason: Sage;2010.

[ref90] McCulloch A : *Spaces and Social Exclusion.* London: Routledge;2005.

[ref58] McKendy J : I’m very careful about that: narrative and agency of men in prison. *Discourse Soc.* 2006;17(4):473–502. 10.1177/0957926506063128

[ref87] McMurran M Khalifa N Gibbons S : *Forensic Mental Health.* Devon: Willan Publishing;2009.

[ref59] Merleau-Ponty M : *Signs. Evanston.* IL: Northwestern University Press;1964.

[ref60] Morgan A Ziglio E : Revitalising the evidence base for public health: an assets model. *Glob. Health Promot.* 2007;14(2, suppl):17–22. 10.1177/10253823070140020701x 17685075

[ref61] Mottershead R : British Military Veterans and the Criminal Justice System in the United Kingdom: Situating the Self in Veteran Research. Unpublished doctoral dissertation. University of Chester, Chester. 2019. Reference Source

[ref63] Mottershead R : The social prescribing of psychosocial interventions in the treatment of addictions and substance use disorders with military veterans: a reclamation of identity and belonging [version 2; peer review: 2 approved]. *F1000Res.* 2022;11:944. 10.12688/f1000research.124768.2 36203746 PMC9513413

[ref64] Mottershead R Ghisoni M : Horticultural therapy, nutrition and post-traumatic stress disorder in post-military veterans: developing non-pharmaceutical interventions to complement existing therapeutic approaches. *F1000Res.* 2021;10:885. 10.12688/f1000research.70643.1 34621518 PMC8456374

[ref65] Mottershead R Alonaizi N : A narrative inquiry into the resettlement of armed forces personnel in the Arabian Gulf: a model for successful transition and positive mental well-being. *F1000Res.* 2021 Dec 16;10:1290. 10.12688/f1000research.75276.1 35035901 PMC8738972

[ref66] Mottershead R Alonaizi N : Empowering social prescribing and peer support: A proposed therapeutic alliance against addictions and substance misuse within the middle east. *J. Drug Alcohol Res.* 2023;11. 10.4303/JDAR/236208

[ref67] Murphy D Palmer E Lock R : Post-traumatic growth among the UK veterans following treatment for post-traumatic stress disorder. *BMJ Mil Health.* 2017;163(2):140–145. 10.1136/jramc-2016-000638 Google Scholar 27451422

[ref68] NAPO: *Armed Forces and the Criminal Justice System.* London: National Association of Probation Officers;2009. (accessed 12.1.23). Reference Source

[ref69] Ochberg RL : *Life Stories and Storied Lives.* Thousand Oakes. CA: Sage;1994.

[ref70] O’Connor M Coleman A : Particularly vulnerable’: homeless young people. *Interaction.* 1995;9(1):8–14.

[ref91] Phillips S : Former members of the armed forces and the criminal just ice system: a review on behalf of the Secretary of State for Justice. 2014. (Accessed: 01.10.2023). Reference Source

[ref71] Plummer K : *Documents of Life 2: An invitation to a Critical Humanism.* Sage Publication;2001. 10.4135/9781849208888

[ref72] Raffo C Dyson A Gunter H : *Education and Poverty in Affluent Countries.* London: Routledge;2011.

[ref73] Ristkari T Sourander A Ronning JA : Sense of coherence and criminal offences among young males. Findings from the Finnish From a Boy to a Man study. *Nord. Psychol.* 2009;61(1):4–13. 10.1027/1901-2276.61.1.4

[ref74] Rosenbaum M Palman N : Helplessness and resourcefulness in coping with epilepsy. *J. Consult. Clin. Psychol.* 1984;52(2):244–253. 10.1037/0022-006X.52.2.244 6715650

[ref75] Royal College of Defence Studies: *The Next Generation of Veterans: Their Critical Needs and Their Emerging Rights.* Royal College of Defence Studies Publication;2009.

[ref76] Savin-Baden M Major CH : *Qualitative research: The essential guide to theory and practice.* Oxon: Routledge;2013.

[ref77] Seligman MEP : Learned helplessness. *Annu. Rev. Med.* 1972;23:407–412. 10.1146/annurev.me.23.020172.002203 4566487

[ref88] Silverman D : *Interpreting Qualitative Data.* 4th Ed. Sage Publications Ltd.;2011.

[ref78] Strumpfer DJW : Salutogenesis: A new paradigm. *S. Afr. J. Psychol.* 1990;20(4):265–276. 10.1177/008124639002000406

[ref79] Tuhus-Dubrow R : *The Power of Positive Deviants: A Promising New Tactic for Changing Communities from the Inside.* The Boston Globe;2009.

[ref80] United Nations Department of Public Information: The universal declaration of human rights. 1948. Reference Source

[ref83] Van Teijlingen E : A Critical Analysis of the Medical Model as used in the Study of Pregnancy and Childbirth. *Sociol. Res. Online.* 2005;10:63–77. 10.5153/sro.1034

[ref84] Williams D : Autoethnography in offender rehabilitation research and practice: addressing the “us vs. them” problem. *Contemp. Justice Rev.* 2006;9(1):23–38. 10.1080/10282580600564818

[ref85] Wortzel HS Binswanger IA Anderson CA : Suicide among incarcerated veterans. *J. Am. Acad. Psychiatry Law.* 2009;37(1):82–91. 19297638

[ref86] Zoellner T Maercker A : Post-traumatic growth in clinical psychology - a critical review and introduction of a two component model. *Clin. Psychol. Rev.* 2006 Sep;26(5):626–653. 10.1016/j.cpr.2006.01.008 16515831

