# Supplementary material for: Salutogenesis: A sense of coherence and health among British military veterans exposed to impactful life challenges - the emergence of post-traumatic growth and positive deviance within a  life-story approach
Source: F1000Res. 2024 Feb 5;13:92. [Version 1] doi: 10.12688/f1000research.145255.1 (PMC10918767; doi:10.12688/f1000research.145255.1)
Supplement: Peer review history

# Reviewer report: Reviewer response for version 1

**DOI:** 10.5256/f1000research.159174.r244667

**Published:** 2024-03-19

**Authors:** Mark Russell

**Affiliations:** 1 Edge Hill University, Ormskirk, England, UK

This research (and associated dissemination) through this paper), presents an interesting and useful exploration of two (sub-)groups of veterans using qualitative methods. The research team set out some useful definitions (Salutogenesis, veteran practitioner / offender) and their research methodology.

 The paper is also usefully located in the literature.

 Conducting seventeen interviews, coding and identifying themes from such interviews is a non-trivial activity. 

 It is noted, and useful, that the paper includes the question set for the two variants of the semi-structured interviews. Such will prove helpful for other researchers wishing to explore similar areas, (and triangulate the findings). Although some questions appear to be ‘closed questions’, I am sure the researcher(s) opened these closed questions during the semi-structured format. I think it would be useful if the authors elaborated  on this point.

 In terms of some opportunities to enhance the paper, unless the current format is a journal requirement, I think the Abstract could / should be crisper and ‘un-sectioned’.

 It would be useful to read more about the process of coding and especially hearing how the researcher’s own life experiences / perception did not bias the codes and themes. I note the paper mentions ‘…research team’s collective analysis of the data.’. **It would be useful to read more about this ‘collective analysis’ process.**

 Linked to the above, it would be useful to read more about the elimination of (recognition of) potential bias and how the research team maintained 'arms length' from the findings. i.e. offer some observations relating to reflexivity.

 Given the veterans' reported shared experience, I would be interested to read if the researchers felt their non-veteran experiences skewed (in any way) the discussions. Simply to avoid any explicit (or implicit) assertions such as, ‘ *you wont understand, you haven’t served*’.

 I note the paper uses...  sense of coherence, Sense of Coherence and SOC. I think a single format would be better.

 To note: The paper does not include the interview transcripts (source data) hence I cannot comment on predictability of conclusions.

Is the work clearly and accurately presented and does it cite the current literature?

Yes

If applicable, is the statistical analysis and its interpretation appropriate?

Not applicable

Are all the source data underlying the results available to ensure full reproducibility?

No

Is the study design appropriate and is the work technically sound?

Yes

Are the conclusions drawn adequately supported by the results?

Yes

Are sufficient details of methods and analysis provided to allow replication by others?

Partly

Reviewer Expertise:

Educational research.

I confirm that I have read this submission and believe that I have an appropriate level of expertise to confirm that it is of an acceptable scientific standard.

# Reviewer report: Reviewer response for version 1

**DOI:** 10.5256/f1000research.159174.r244664

**Published:** 2024-03-06

**Authors:** John Williams

**Affiliations:** 1 Aberystwyth University, Aberystwyth, Wales, UK

The article explores the sense of coherence and health among British military veterans who have experienced significant life challenges, focusing on the emergence of post-traumatic growth and positive deviance within a life-story approach.

 It provides valuable insight into two groups of military veterans in the United Kingdom, namely those identified as offenders and those working within the criminal justice system. It adopts a salutogenic approach that emphasises resilience and reconstructing military experience to enable a sense of coherence. The research was conducted based on the first author's PhD thesis and thus has a robust academic approach; it relies on a variety of sources, including the appropriate literature in monographs, journal articles, and reports. Qualitative research methodologies such as narrative inquiry and thematic analysis are employed in the study, delving into issues such as military innovation, health and coping, post-traumatic growth, and the criminal justice system's treatment of veterans.

 The study's findings are valuable as they present evidence of post-traumatic growth and positive deviation within the veterans' life stories, highlighting the significance of veteran identity and culture and the bond and support within the veteran community. The participants in the research displayed improvisation, adaptation, and problem-solving skills, establishing new systems and initiatives to support fellow veterans within the criminal justice system. Internal, social and community resources all played a part in improving their lives and the lives of other veterans.

 Overall, the research highlights the resilience and determination of the participants who contributed to the study. The research acknowledges the adversities veterans have faced and are still facing and the need to continue providing them with the support and resources they need to ensure their well-being.

 An interesting piece of research, the findings of which will help the academic community and others to better understand the complexities experienced by veterans and it provides a good foundation for ongoing work with this group.

Is the work clearly and accurately presented and does it cite the current literature?

Yes

If applicable, is the statistical analysis and its interpretation appropriate?

I cannot comment. A qualified statistician is required.

Are all the source data underlying the results available to ensure full reproducibility?

Yes

Is the study design appropriate and is the work technically sound?

Yes

Are the conclusions drawn adequately supported by the results?

Yes

Are sufficient details of methods and analysis provided to allow replication by others?

Yes

Reviewer Expertise:

Vulnerable people and the law. Human Rights.

I confirm that I have read this submission and believe that I have an appropriate level of expertise to confirm that it is of an acceptable scientific standard.

# Reviewer report: Reviewer response for version 1

**DOI:** 10.5256/f1000research.159174.r244671

**Published:** 2024-03-06

**Authors:** Adam Mahmoud Salameh Khraisat

**Affiliations:** 1 Higher Colleges of Technology, Abu Dhabi, United Arab Emirates

This article delves into the experiences and perspectives of two distinct groups within the UK military veteran community: those who have had encounters with the criminal justice system and those working within the criminal justice system. The study utilizes narrative inquiry and life story research, conducting 17 in-depth interviews across England and Wales. The aim is to explore the concept of salutogenesis, which focuses on how individuals maintain health and well-being despite adverse conditions, particularly in the context of military transition. The findings suggest that health cannot be narrowly defined and that various factors, including social, psychological, and spiritual well-being, contribute to resilience and overall health outcomes.

**Major Points:**

Strengths:

- Comprehensive Exploration: The article thoroughly explores the concept of salutogenesis and its application to understanding the health of military veterans. It delves into various dimensions of health, including physical, psychological, spiritual, and social well-being, providing a holistic perspective.
- Inclusive Methodology: The use of narrative inquiry and life story research allows for a deep understanding of participants' lived experiences, capturing diverse perspectives within the veteran community.
- Theoretical Integration: The article effectively integrates theoretical frameworks such as salutogenesis, Sense of Coherence (SOC), and biopsychosocial models to analyze health outcomes, offering a nuanced understanding of factors contributing to resilience and well-being.

Weaknesses: 

- Limited Generalizability: The study's sample size is relatively small, comprising only 17 participants, which may limit the generalizability of findings to the broader veteran population. Additionally, the focus on UK military veterans and practitioners within the criminal justice system may restrict the applicability of findings to other contexts.
- Potential Bias: As the lead author is a health professional, there might be a bias towards health-centric perspectives, potentially overlooking other important factors influencing veterans' experiences and well-being. and as I mentioned previously, the results should be given to another Qualitative data analyzer to check the codes and themes building process and maintain the trustworthiness of this article's findings. 
- Lack of Discussion on Methodological Limitations: The article lacks a discussion on potential limitations of the methodology employed, such as selection bias, interviewer bias, or the influence of social desirability on participants' responses.

**Minor Points: **

- Clarity and Organization: The article could benefit from clearer organization and structure, particularly in presenting the findings and discussing their implications. Clear subsections or headings could enhance readability and comprehension.
- Integration of Additional Literature: While the article extensively discusses salutogenesis and related concepts, it could further enrich the discussion by integrating findings from other relevant literature, particularly studies focusing on veterans' experiences and transition challenges.

Overall, the article offers valuable insights into the complex interplay between health, resilience, and lived experiences among military veterans and practitioners within the criminal justice system. However, addressing the identified weaknesses could strengthen the robustness and applicability of the study's findings.

Is the work clearly and accurately presented and does it cite the current literature?

Yes

If applicable, is the statistical analysis and its interpretation appropriate?

Not applicable

Are all the source data underlying the results available to ensure full reproducibility?

Yes

Is the study design appropriate and is the work technically sound?

Yes

Are the conclusions drawn adequately supported by the results?

Yes

Are sufficient details of methods and analysis provided to allow replication by others?

Partly

Reviewer Expertise:

Emotional intelligence, Qualitative data analysis, mixed methods research, Nursing Education,

I confirm that I have read this submission and believe that I have an appropriate level of expertise to confirm that it is of an acceptable scientific standard.

# Reviewer report: Reviewer response for version 1

**DOI:** 10.5256/f1000research.159174.r244666

**Published:** 2024-03-06

**Authors:** David Jackson

**Affiliations:** 1 Exeter University, Exeter, UK

This research looked at the life experiences and the health of two subgroups of veterans. There were 17 participants 8 who were practitioners within the criminal justice system and 7 who were defined as offenders. They all identified as military veterans. The study explored health issues within the trajectory of their lives using life story methods.  It found that there was a process of adaptation through the identity of being a veteran. This was found to be an ongoing and dynamic process. The study suggests that the development of a sense of coherence is ongoing through life trajectories. It is achieved through post-traumatic growth, positive deviance and an identification with their past lives of military service and life.

 As a researcher who is also a veteran and whose research has extensively used life story methods and thematic analysis, I think this paper is excellent. It contributes to the growth in research that offers a voice and gives agency to the veteran community. For example, the Military Afterlives ( Military Afterlives Project) and Stories in Transition Project ( Stories in Transition). The reasons for using life story and thematic analysis were very clear and well-argued citing some important published work in this area.  The method section showed rigour and clarity. I really liked the contextualisation of salutogenesis and the importance placed upon the sense of coherence and general resistance resources theoretical frameworks. The important point being that these veterans’ life experiences are not lived out within a vacuum. The authors findings from his interviews raise some very important points in relation to the notion of veteran/military identity and a connection with past military experience. It is an important part of having a positive veteran self-identity. It was alluded to by the participants that society did not fully comprehend them and their experiences. I would add to this point that society does comprehend veterans but are informed by historical and cultural assumed knowledge (Armour, C. *et al*, 2018 (Ref 1), Ashcroft, M., & KCMG 20149 ( 68364 The Armed Forces and Society.pdf (lordashcroftpolls.com), You.Gov 2018). Secondly, there is potential in the future for further discussions to consider insider/outsider research. There is a growing cohort of researchers who are veterans researching the veteran community. Whilst the author acknowledged his military identity at the end of the paper and recognised  possible bias. I think there is an opportunity to explore the benefits of ‘insiders’ conducting veteran research more especially in relation to the comradeship and commonality of experience felt within the veteran community.  How does "the space between" insider and outsider researchers differ when participating in veteran research? (Kukutai, T. *et.al,* 2011 (Ref 2)

 I really enjoyed reading this.

Is the work clearly and accurately presented and does it cite the current literature?

Yes

If applicable, is the statistical analysis and its interpretation appropriate?

Yes

Are all the source data underlying the results available to ensure full reproducibility?

Yes

Is the study design appropriate and is the work technically sound?

Yes

Are the conclusions drawn adequately supported by the results?

Yes

Are sufficient details of methods and analysis provided to allow replication by others?

Yes

Reviewer Expertise:

Veteran research, Creative Methods, Life story Narrative, autoethnography, Multi modal representations of narrative

I confirm that I have read this submission and believe that I have an appropriate level of expertise to confirm that it is of an acceptable scientific standard.

## References

[1] : Public Attitudes to the UK Armed Forces in Northern Ireland.2018;

[2] : Navigating the 'space between': Authenticity and identity in 'māori' social science.2011;26:4-20
